# A Pilot Proteomics Analysis of *Mycobacterium tuberculosis* Lacking a Putative Short-Chain Dehydrogenase Rv0148

**DOI:** 10.3390/ijms27188318

**Published:** 2026-09-18

**Authors:** Gunapati Bhargavi, Anbarasu Deenadayalan, Kannan Palaniyandi, Selvakumar Subbian

**Affiliations:** 1 Department of Immunology, Indian Council of Medical Research-National Institute for Research in Tuberculosis, Chetpet, Chennai 600031, India; gb603@njms.rutgers.edu (G.B.); harianbu@nirt.res.in (A.D.); 2 Public Health Research Institute, New Jersey Medical School, Rutgers University, Newark, NJ 07103, USA

**Keywords:** mycobacterium, capsule, cell wall, metabolism, immune response, peptidoglycan, stress adaptation, STRING analysis, protein–protein interactions, LC/MS analysis

## Abstract

*Mycobacterium tuberculosis* (*Mtb*) is an intracellular pathogen that survives in host cells by resisting hostile antimicrobial defenses. However, the molecules and mechanisms that contribute to *Mtb*’s intracellular survival are not fully understood. We have previously reported that Rv0148, a putative short-chain dehydrogenase/reductase, plays a significant role in *Mtb* stress response and virulence in in vitro and in vivo models. To further understand the role of Rv0148 in regulating global functions of *Mtb*, we performed comparative pilot proteomic analysis between *Δrv0148* mutant, and wild-type (WT) strains grown in axenic cultures. Mass spectrometry-based proteomics analysis identified 586 and 626 statistically significant differentially abundant proteins (SSDAP) in the WT and *Δrv0148* mutant, respectively. Ontology analysis revealed that proteins involved in biological processes, including bacterial adaptation to host responses and protein homeostasis, were significantly more abundant, while peptidoglycan biosynthesis was less abundant in the mutant than the WT. Further network analysis revealed dysregulation of proteins involved in bacterial stress response, cell wall components, ribosomal and secretory proteins, suggesting impaired cell wall and translation machinery in *Δrv0148.* Functional categorization of differentially regulated proteins in *Δrv0148* showed broad reprogramming in intermediary metabolism and stress adaptation. These findings suggest that Rv0148 may influence remodeling of cell wall components and bacterial physiology, potentially balancing *Mtb* survival and stress adaptation.

## 1. Introduction

Tuberculosis (TB), caused by *Mycobacterium tuberculosis* (*Mtb*), remains the leading cause of death from a single infectious disease worldwide. The World Health Organization (WHO) estimated 10.7 million new TB cases and 1.23 million TB-related deaths globally in 2024 [1]. The pathogenesis of TB is driven by complex host–pathogen interactions occurring both at the site of infection and systemically, resulting in a spectrum of clinical outcomes ranging from asymptomatic latent *Mtb* infection (LTBI) to active symptomatic disease [2,3]. Shortly after entering the lungs, *Mtb* is engulfed by host antigen-presenting cells (APCs), such as macrophages, which attempt to eliminate the pathogen through various antimicrobial mechanisms, including the production of reactive oxygen and nitrogen species (ROS and RNS) [4,5,6]. However, *Mtb* can evade these antimicrobial responses and replicate intracellularly through metabolic and structural adaptations that promote its survival [7,8]. For example, studies of the DosR regulon, which comprises genes regulated by the DosR protein in response to hypoxia and exposure to ROS and RNS, have provided important insights into the mechanisms that enable *Mtb* to survive in the oxygen-limited and stressful environment of granulomas, thereby improving our understanding of TB pathogenesis [9,10,11,12].

In addition to canonical stress-responsive proteins such as DosR, several other *Mtb* proteins, including members of the short-chain dehydrogenase/reductase (SDR) family, play important roles in cellular metabolism and redox homeostasis [13,14]. SDRs constitute a diverse group of NAD(P)(H)-dependent oxidoreductases that participate in a wide range of metabolic pathways and contribute to the ability of *Mtb* to adapt to hostile host environments [15,16]. Recent studies indicate that SDR enzymes are involved in protecting *Mtb* against ROS and RNS stress, thereby promoting bacterial survival during infection [15,16]. Previously, we reported that *rv0148* is an SDR that plays an important role in *Mtb* survival and persistence within the host by influencing bacterial stress management and immune evasion [15,16]. We demonstrated that, compared with the wild type (WT), the *Δrv0148* strain was significantly more susceptible to killing by a variety of stress-inducing agents in vitro, including electron chain inhibitors (rotenone, oxaloacetate), H_2_O_2_, Cumene hydroperoxide, DTT, and DMSO. Furthermore, the *Δrv0148* strain was attenuated in virulence and showed reduced intracellular survival in macrophages as well as in the lungs and spleens of infected guinea pigs [15,16]. In addition, macrophages infected with the *Δrv0148* strain expressed elevated levels of pro-inflammatory cytokines, including TNF-α and IL-1β, compared with those infected with the WT, suggesting that Rv0148 may contribute to modulation of host immune responses [15,16,17]. However, global proteomic characterization of protein abundance in the *Δrv0148* mutant relative to the WT strain has not yet been reported.

Proteomics, a comprehensive approach for large-scale analysis of protein abundance, has emerged as a powerful tool for elucidating the molecular mechanisms underlying TB pathogenesis [18,19,20,21,22]. By enabling the identification and quantification of large numbers of proteins and their interaction networks, proteomics provides insights into host–*Mtb* interactions and the bacterial adaptation strategies that facilitate survival, replication, and persistence within the hostile host environment [18,19,20,21,22].

Building on our previous findings, we applied a mass spectrometry-based proteomics analysis in this exploratory study to determine the changes in global protein levels in *Δrv0148* compared with the WT strain. Our preliminary analyses revealed altered abundance of proteins associated with ribosomal function, protein secretion systems, stress responses, protein acetylation, and peptidoglycan biosynthesis in *Δrv0148* compared to the WT strain. Collectively, these findings underscore the multifaceted role of Rv0148 in regulating *Mtb* stress responses, protein secretion, and cellular homeostasis.

## 2. Results

### 2.1. Comparative Proteomic Analysis of WT H37Rv and Δrv0148 Strains

**Figure 1:**
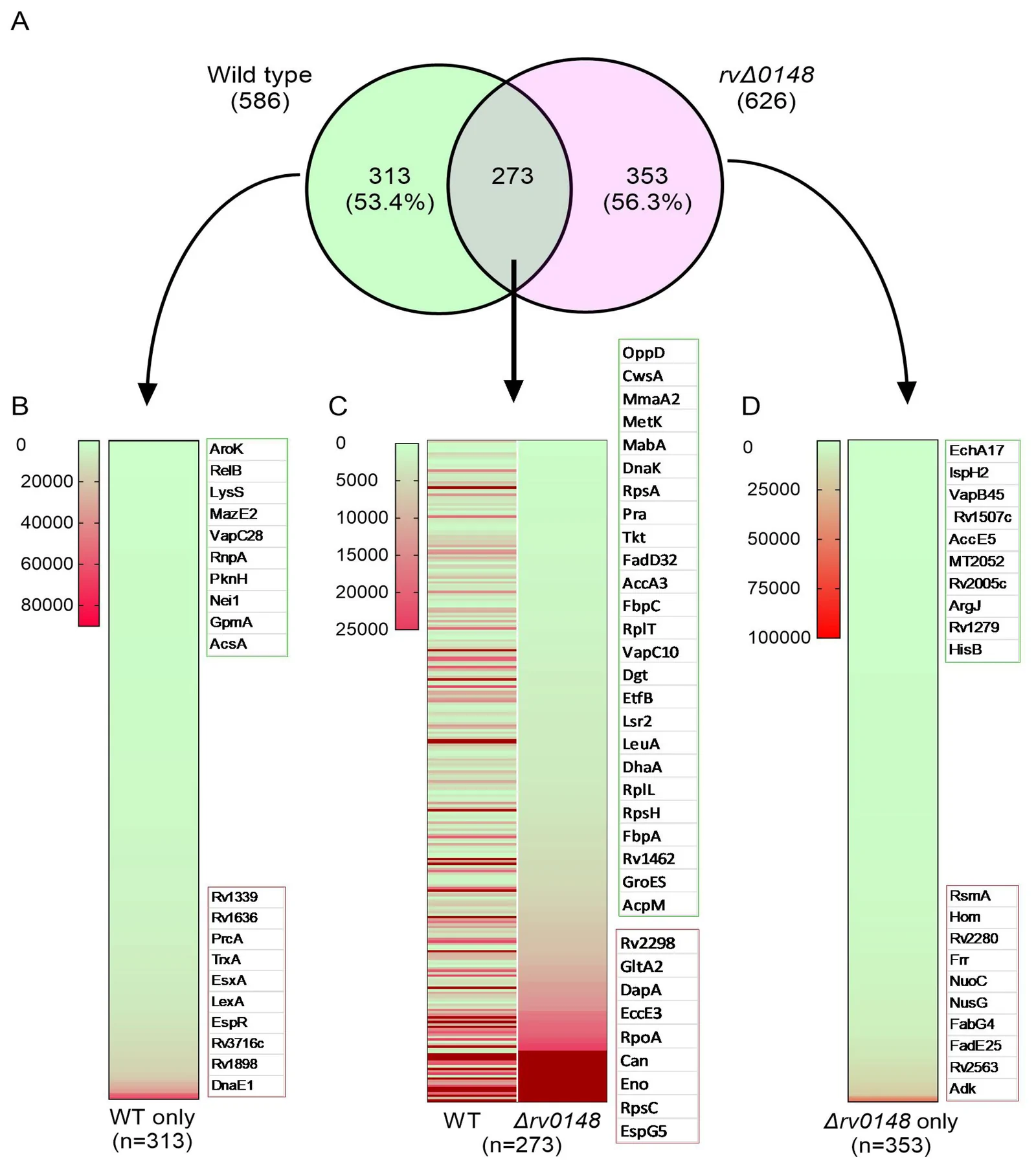
Overview of comparative proteomics of WT and *Δrv0148* strains. (**A**).Venn diagram showing the number of unique and shared SSDAPs between the WT and *Δrv0148* mutant. A total of 939 SSDAPs were detected, which includes 313 and 353 proteins exclusively detected in the WT and*Δrv0148* strains, respectively, and 273 proteins common to both strains; these protein identification codes were verified with the UniProt *Mtb* database. (**B**). Normalized abundance value-based heatmap showing the abundance profile of 313 SSDAPs detected only in the WT. The top 10 most and least abundant proteins are listed in red and green boxes, respectively. (**C**). Heatmap showing the abundance profile of 273 SSDAPs detected both in the WT and *Δrv0148* strains. SSDAPs that are more or less abundant in *Δrv0148* compared to the WT are listed in red and green boxes, respectively. (**D**). Heatmap showing the abundance profile of 353 SSDAPs detected only in *Δrv0148*. The top 10 most and least abundant proteins are listed in red and green boxes, respectively. For each identified and annotated *Mtb* protein, identification codes were verified with the UniProt MtbH37Rv database, and the normalized abundance value was determined using a false discovery rate (FDR) cutoff of 4%. Heat maps for (**B**) the WT, (**C**) both the WT and *Δ0148*, and (**D**) *Δ0148* were plotted using the normalized abundance values. The scale bar in (**B**–**D**) corresponds to normalized abundance values from least abundant (green) to most abundant (red) values. The heatmap was formatted from low (top; green) to high (bottom; red) normalized abundance, and the images were created using GraphPad Prism 10.

To assess the impact of *rv0148* deletion on the abundance of *Mtb* proteins, we performed a comparative proteomic analysis between the WT and *Δrv0148* mutant strains. As shown in Figure 1A and Supplementary Table S1, a total of 939 statistically significant differentially abundant proteins (SSDAPs) were identified across the two strains. Among these, 353 proteins were detected exclusively in *Δrv0148*, whereas 313 proteins were detected only in the WT strain. An additional 273 proteins were detected in both strains (Figure 1A).

**Figure 2:**
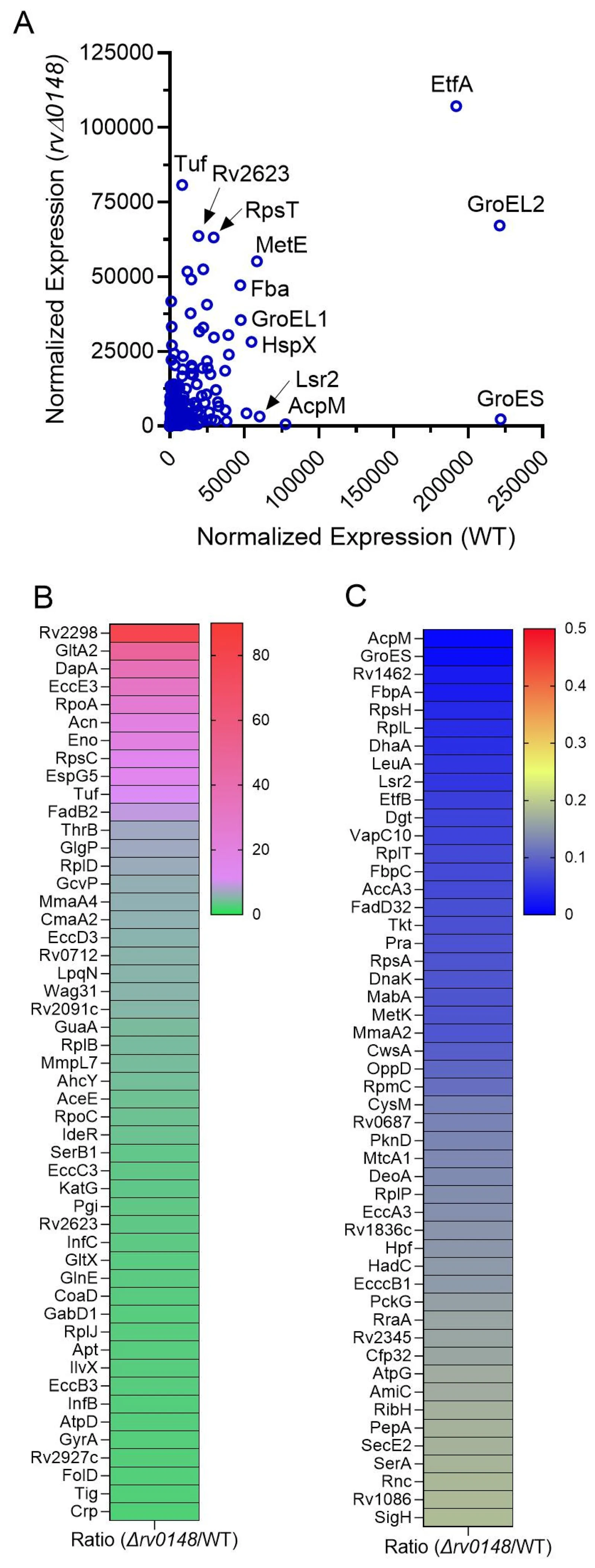
Profile of SSDAPs in the WT and *Δrv0148* mutant strains. (**A**). Scatter plot showing the abundance patten of proteins detected in both WT and *Δrv0148* strains. Each point represents the group-level summary of normalized abundance values of proteins in *Δrv0148* (*y*-axis) and its corresponding value in the WT strain (*x*-axis). The plot illustrates the overall distribution of SSDAPs based on normalized abundance values, highlighting proteins that are more abundant in the WT or *Δrv0148* strains (**B**). Heatmap of top 50 most abundant proteins in *Δrv0148*, relative to the WT strain. (**C**). Heatmap of top 50 least abundant proteins in *Δrv0148*, relative to the WT strain. The ratio of protein abundance in *Δrv0148* compared to the WT strain was used to generate the heat map. Color intensity reflects the fold change in abundance, with the gradient scale indicating increasing abundance from green/pink/red (**B**) or blue/yellow/red (**C**). The scatter plot and heatmaps were created using GraphPad Prism 10.

Distinct abundance patterns were observed among the common SSDAPs detected both in the WT and *Δrv0148* strains, based on the normalized abundance values (Figure 1B–D and Figure 2A). Compared to the WT, 8 proteins (Rv2298, GltA2, DapA, RpoA, Acn, Eno, RpsC, and EspG5) were more abundant, while 25 proteins (AcpM, GroES, Rv1462, FbpA, RpsH, RplL, DhaA, LeuA, Lsr2, EtfB, Dgt, VapC10, RplT, FbpC, AccA3, FadD32, Tkt, Pra, RpsA, DnaK, MabA, MetK, MmaA2, CwsA, and OppD) were less abundant by more than 10-fold in the mutant strain (Figure 2B). The identities and predicted functions of these SSDAPs suggest that the *Δrv0148* strain undergoes extensive physiological and metabolic remodeling. This remodeling is characterized by reduced abundance of proteins involved in cell wall and mycolic acid biosynthesis (e.g., AcpM, AccA3, FadD32, MabA, MmaA2, FbpA, and FbpC), as well as protein translation (e.g., RpsH, RplL, RplT). In contrast, increased abundance of proteins associated with central carbon metabolism (e.g., GltA2, Acn, and Eno), transcriptional regulation (e.g., RpoA), and some of the secretion system components (e.g., EccB3 and EspB) suggests metabolic reprogramming and activation of compensatory pathways. Together, these findings indicate that Rv0148 may play an important role in coordinating *Mtb* metabolism and cell wall synthesis and may consequently influence bacterial fitness and virulence.

### 2.2. GO Analysis of More Abundant SSDAPs in the Δrv0148 Mutant

**Figure 3:**
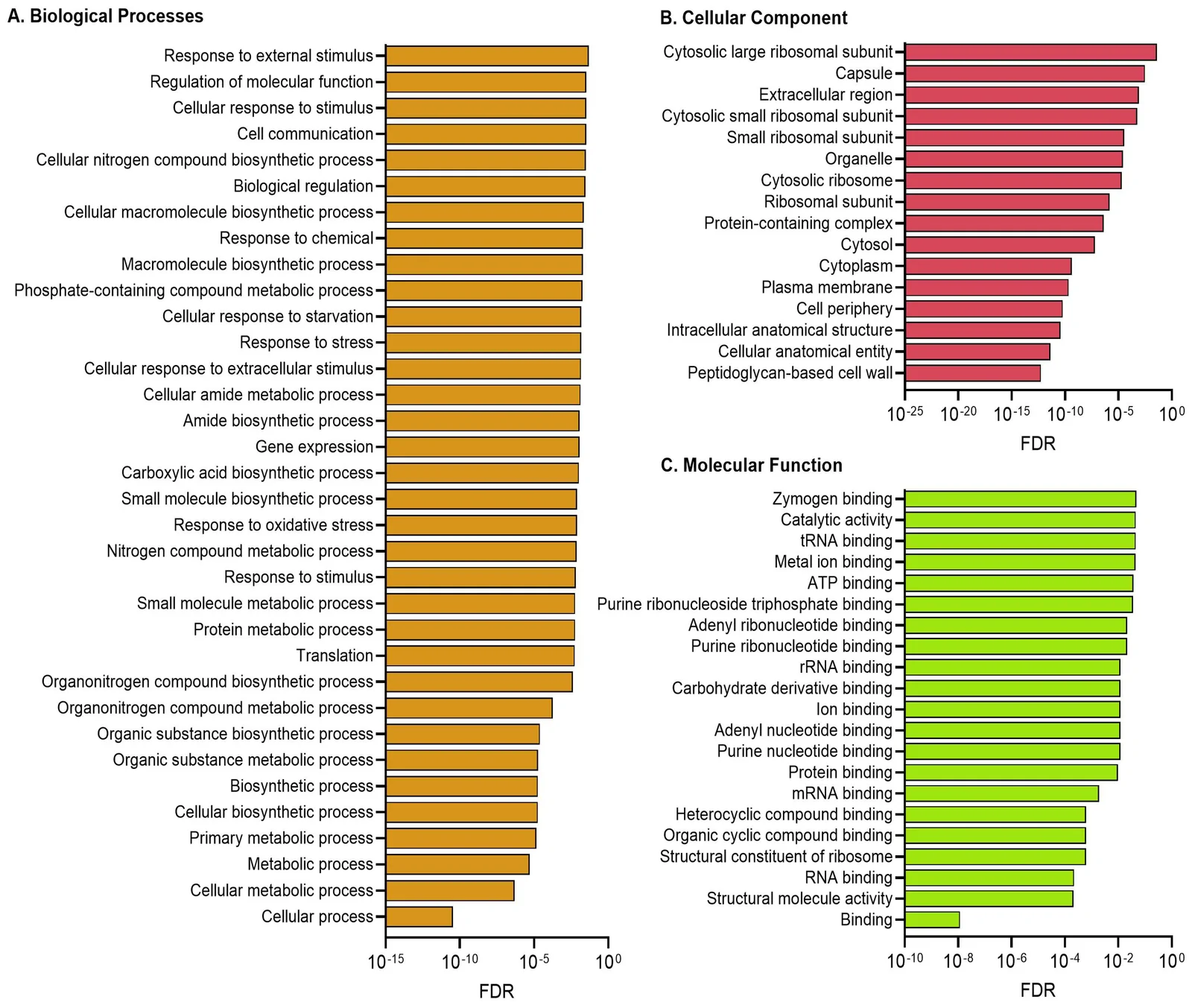
GO enrichment analysis of more abundant SSDAPs in *Δrv0148* compared to the WT. The bar plots show enriched GO categories in biological processes (**A**). cellular components (**B**). and molecular functions (**C**). Enrichment analysis was performed using more abundant proteins in *Δrv0148* relative to the WT strain. The *x*-axis represents the FDR, plotted on a log scale. Lower FDR values indicate higher statistical significance of enrichment. The bar graphs were created using GraphPad Prism 10.

To further investigate the functional significance of the most abundant SSDAPs in *Δrv0148*, compared to the WT strain, we performed Gene Ontology (GO) analysis using ShinyGO. The significantly enriched GO terms (FDR < 0.05) were categorized into three main domains: (i) biological processes, (ii) cellular compartments, and (iii) molecular functions (Figure 3).

Within the biological processes category, 48 significantly enriched GO terms, including overlapping parent and child terms, were identified (Figure 3A). Notably, these included GO terms associated with stress response and virulence, cellular response to starvation, and response to chemical or cellular stimuli. Enrichment of these pathways suggests that deletion of Rv0148 affects the adaptive mechanisms that *Mtb* utilizes to survive hostile conditions encountered within the host. Additional GO-enriched terms, such as protein folding, cell communication, cellular nitrogen compound metabolic process, and regulation of primary metabolic process, indicate a broad shift in cellular reprogramming in *Δrv0148*. Together, these changes suggest an increased emphasis on maintaining protein homeostasis and adapting metabolic functions in response to the loss of Rv0148 (Figure 3A).

Analysis of the cellular component category revealed that the more abundant SSDAPs in *Δrv0148* were predominantly associated with the plasma membrane, protein-containing complexes, nucleoid, cytosol, and intracellular non-membrane-bounded organelles (Figure 3B). In the molecular function category, more abundant SSDAPs are associated with binding and catalytic enzyme activities (Figure 3C). Specifically, functions such as adenylyl transferase activity, unfolded protein binding, rRNA binding, ATP binding, and nucleoside–triphosphatase activity were significantly overrepresented. These findings suggest enhanced activity of pathways involved in nucleotide metabolism and ribonucleotide processing, which may support the adaptive response of *Δrv0148*. Importantly, interaction network analysis revealed extensive connectivity among the more abundant SSDAP, highlighting coordinated regulation of multiple biological pathways disrupted in the *Δrv0148* (Supplementary Figure S1A). These interconnected networks further support the notion that loss of Rv0148 triggers a broad adaptive response involving stress adaptation, metabolic reprogramming, and maintenance of cellular homeostasis.

### 2.3. GO Analysis of Less Abundant SSDAPs in Δrv0148

**Figure 4:**
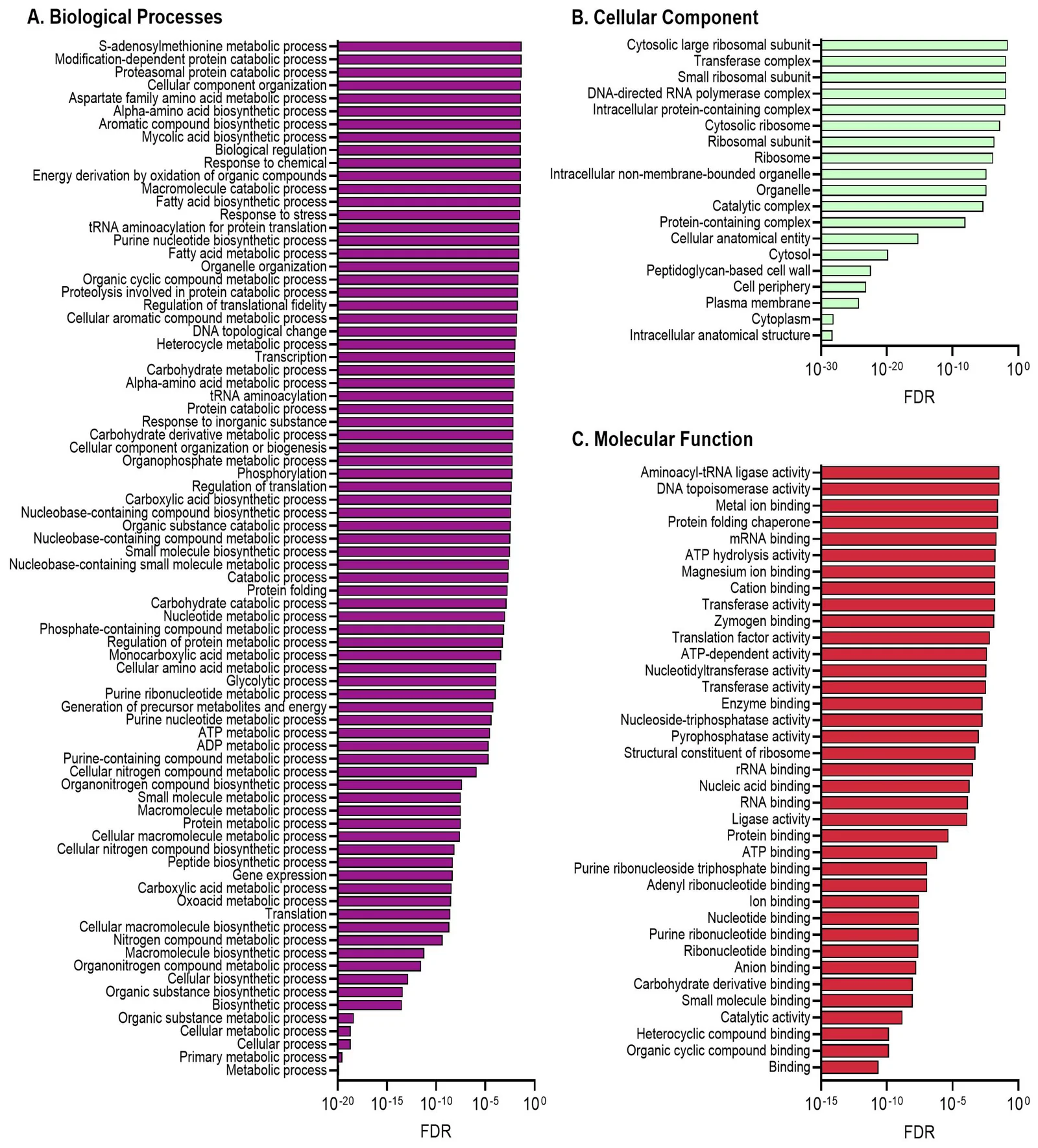
GO enrichment analysis of less abundant SSDAPs in *Δrv0148* compared to the WT strain. The bar plots show enriched GO categories in biological processes (**A**). cellular components (**B**). and molecular functions (**C**). Enrichment analysis was performed using less abundant proteins in *Δrv0148* relative to the WT strain. The *x*-axis represents the FDR, plotted on a log scale. Lower FDR values indicate higher statistical significance of enrichment. The bar graphs were created using GraphPad Prism 10.

A total of 117 less abundant SSDAPs were analyzed for GO. Among these, a wide range of biological functions were associated with less abundant SSDAPs in *Δrv0148* compared to the WT strain, particularly those related to biosynthesis and cellular regulation (Figure 4A). The most significantly enriched GO terms within the biological process category included regulation of carboxylic acid metabolism, cellular response to stress, and negative regulation of macromolecule biosynthetic processes. In addition, GO terms associated with translation, protein metabolism, gene expression, cellular homeostasis, and nitrogen compound biosynthesis were significantly enriched among the less abundant SSDAPs. Collectively, these findings indicate a widespread alteration in stress response pathways and core metabolic functions in *Δrv0148*, compared to the WT strain.

Analysis of the cellular component category revealed that many of the less abundant SSDAPs were associated with ribosomal structures, including the large and small ribosomal subunits, cytosolic ribosomes, protein-containing complexes, the cytosol, and intracellular organelles (Figure 4B). This enrichment pattern suggests impaired ribosome biogenesis and reduced translational capacity in the *Δrv0148* mutant. Additional enrichment was observed for proteins associated with the extracellular region, plasma membrane, and cell periphery, indicating broader effects on cell envelope organization and potentially on protein secretion pathways.

Within the molecular function category, the SSDAP profile indicates that catalytic activity, metal ion binding, ATP binding, and structural molecule activity were dampened (Figure 4C). Several proteins associated with biomolecule-binding functions, including RNA binding and heterocyclic compound binding, were also reduced in abundance. These patterns suggest altered enzymatic activity, oxidative stress defense mechanisms, and nucleic acid-associated functions in *Δrv0148* compared to the WT strain. Furthermore, protein interaction network analysis revealed extensive connectivity among the less abundant SSDAPs, highlighting coordinated disruption of multiple biological pathways in the mutant (Supplementary Figure S1B).

### 2.4. Functional Categorization of SSDAPs Detected in Δrv0148

**Figure 5:**
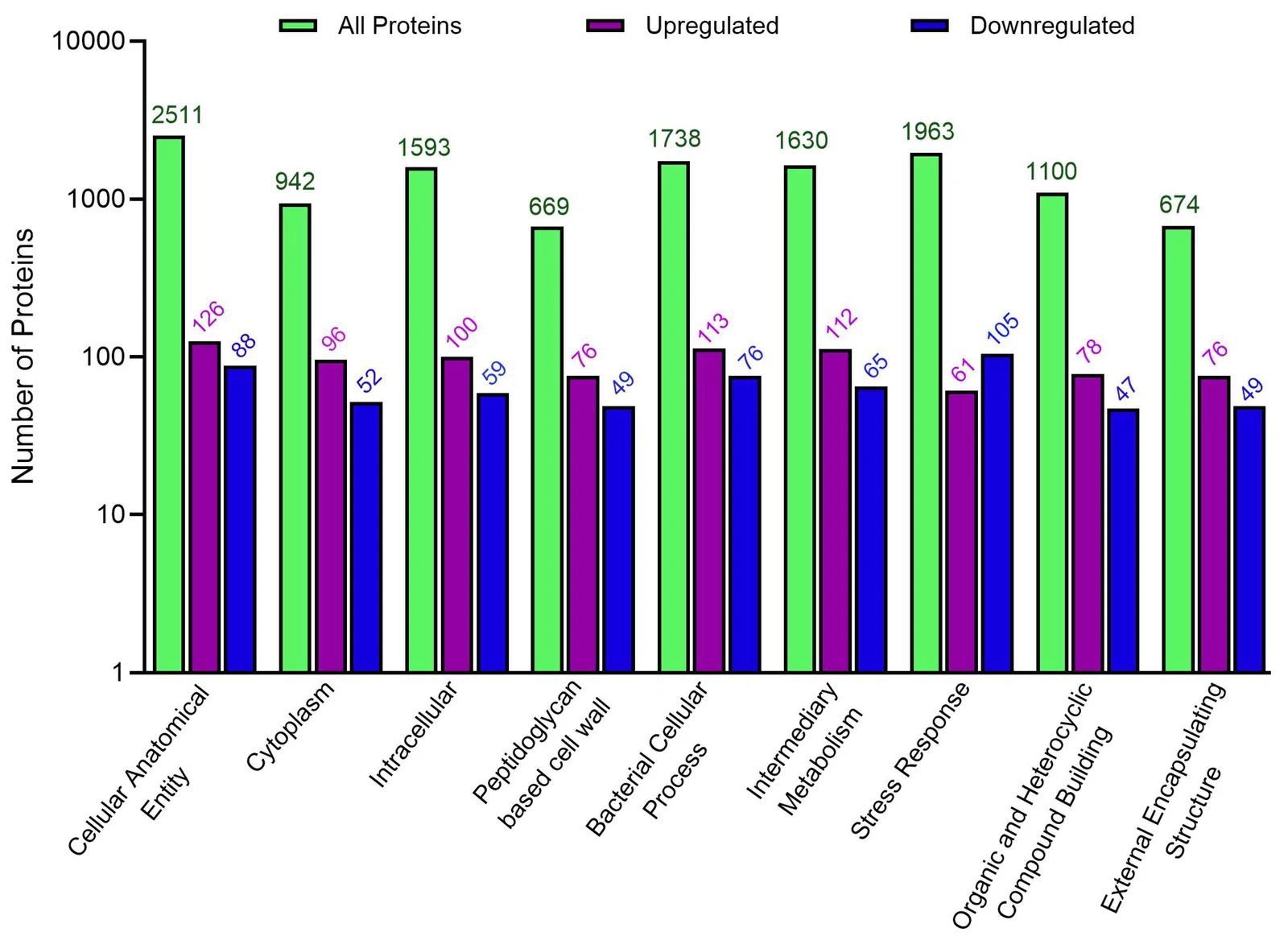
Functional classification of SSDAPs enriched in *Δrv0148*. The bar chart displays the distribution of SSDAPs across various functional categories in *Δrv0148*, compared to in the WT strain. The total number of detected proteins in each functional category is shown in green (total proteins in a specific GO category are marked as all proteins). The numbers of the most and least abundant proteins in *Δrv0148* are shown in purple and blue colors, respectively. Data analysis for functional classification was defined and assigned automatically based on Mycobrowser *Mtb*H37Rv annotation and the STRING 12.0 database. The bar graph was created using GraphPad Prism 10.

To further contextualize the biological significance of SSDAPs in *Δrv0148*, we performed GO-based functional categorization analysis (Figure 5). Among the functional categories in ShinyGo, cellular anatomical entities contained the largest number of SSDAPs, including 126 more abundant and 88 less abundant proteins. Consistently, 96 more abundant and 52 less abundant proteins were localized to the cytoplasm, whereas 100 more abundant and 59 less abundant proteins were associated with the intracellular regions. These observations suggest that Rv0148 influences protein localization and structural organization within *Mtb*.

A substantial number of SSDAPs were associated with intermediary metabolism (112 more abundant and 65 less abundant proteins), indicating broad metabolic perturbation following Rv0148 deletion. Particularly, proteins associated with peptidoglycan biosynthesis and cell wall-associated processes were more abundant (76 out of 125), suggesting alterations in cell wall composition and remodeling. Such changes may contribute to the altered ability of *Δrv0148* to adapt to stress and antimicrobial pressure.

A similar trend was also observed for proteins involved in organic and heterocyclic compound building-related proteins (78 more abundant protein out of 125), suggesting adaptation to stress response through modulation of bacterial structural components (Figure 5). Finally, proteins involved in *Mtb* cellular processes showed marked differential abundance with 113 more abundant and 76 less abundant in *Δrv0148.* These findings are consistent with the STRING network analysis, which indicated altered regulation of components associated with the ESX-1 secretion systems. Further, proteins involved in translation and protein folding also showed differential abundance. Notably, molecular chaperones such as GroES and GroEL1, which are known to be induced during heat shock and oxidative stress, were more abundant in *Δrv0148* [23,24]. Taken together, these results suggest that deletion of *rv0148* impacts multiple interconnected pathways in *Mtb*, notably those involved in metabolism, adaptation to stress, and protein homeostasis.

### 2.5. STRING Analysis of Highly Enriched Networks in the Δrv0148 Mutant

**Figure 6:**
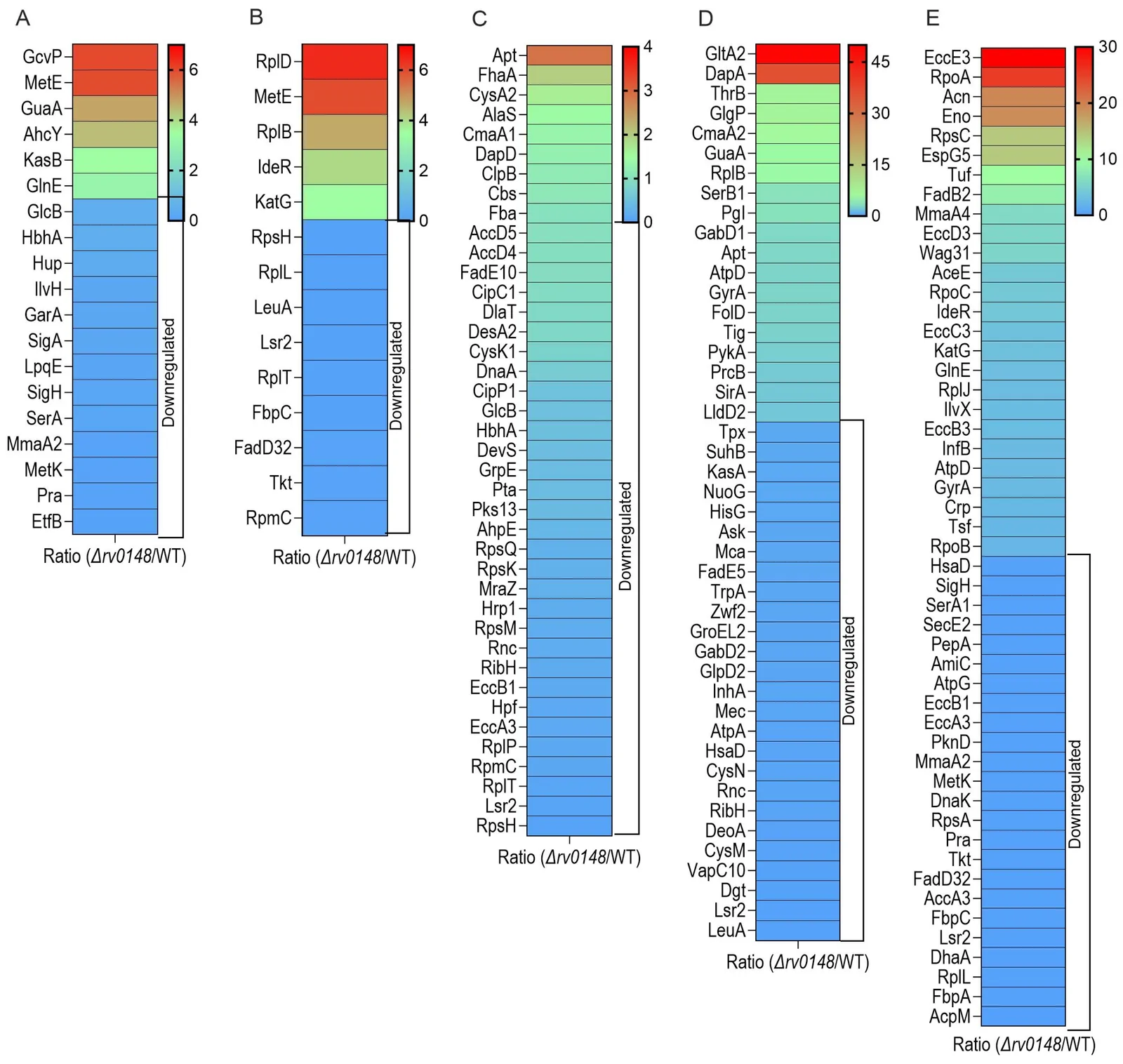
Selected pathway analysis of SSDAPs in *Δrv0148*. The ratio of SSDAPs detected between *Δrv0148* and the WT strains was used to generate the heat maps in (**A**–**E**). Specific pathways include external capsule structure (**A**). intermediary metabolism (**B**). cytoplasmic localization (**C**). stress response (**D**). and peptidoglycan metabolism (**E**). STRING 12.0, with organism *Mtb* H37Rv as a background was assigned automatically, with a high confidence score set at 0.7 (high confidence) and FDR < 0.05. To maintain consistency, the disconnected/orphan nodes were removed. Scale bars represent the abundant ratio (mutant/WT). The color intensity represents the highest (red color) or lowest (blue color) abundance ratio in the *Δrv0148* mutant. Less abundant proteins are marked with boxes in each of the heat maps. The heatmaps were created using GraphPad Prism 10.

To further define protein networks associated with the *Δrv0148* phenotype, we performed protein–protein interaction (PPI) analysis of SSDAPs using STRING (Figure 6 and Figure S2). The major functional clusters identified included cell envelope organization, intermediary metabolism, cytoplasmic proteins, stress response pathways, ribosomal proteins, and peptidoglycan metabolism. These networks were also among the most significantly enriched categories identified in the functional categorization analysis, as shown in Figure 5.

(a) Cell Envelope and Capsular Structure Network: Proteomic analysis revealed 19 SSDAPs associated with the outer capsular and cell envelope structures in *Δrv0148*, compared to the WT strain. Six of these proteins (GcvP-6.0, MetE-5.9, GuaA-4.7, AhcY-4.3, KasB-3.4, and GlnE-3.0) were abundant by more than twofold. In contrast, thirteen proteins (GlcB-0.49, HbhA-0.48, HupB-0.38, IlvH-0.24, GarA-0.24, SigA-0.23, LpqE-0.20, SigH-0.18, SerA-0.17, MmaA2-0.08, MetK-0.08, PraA-0.08, and EtfB-0.06) were less abundant by more than twofold, compared with the WT strain (Figure 6A). While more abundant proteins such as KasB may reflect compensatory responses aimed at preserving cell envelope integrity, less abundant proteins such as an alternative sigma factor (SigH), cell wall lipoprotein (LpqE), and a methyltransferase responsible for the cyclopropanation of mycolic acids (MmaA2) may suggest altered regulation of cell surface lipids in order to cope with host-induced stress. Together, the SSDAP profile suggests a potential role for Rv0148 in cell envelope- and virulence-associated pathways in *Mtb*.
(b) Intermediary Metabolism Network: Within this network, the metal-responsive regulator IdeR and metabolic enzyme MetE were three times more abundant in *Δrv0148* than in the WT strain. Additional proteins, including the 50S ribosomal proteins RplD and RplB and catalase–peroxidase (KatG), were two times more abundant. Conversely, other proteins such as RpsH, RplL, RplT, RpmC, LeuA, Lsr2, FbpC, FadD32 and Tkt were less abundant (Figure 6B), suggesting potential perturbations in protein synthesis, lipid metabolism, and central metabolic pathways in *Δrv0148*.
(c) Cytoplasmic Protein Network: Compared to the WT, the *Δrv0148* strain exhibited alterations in cytoplasmic protein abundance (Figure 6C). For example, proteins including Apt, FhaA, CysA2, AlaS, CmaA1, DapD, ClpB, and Cbs were more abundant relative to the WT strain. In contrast, several ribosome-associated proteins, including RpmC, RplT, RpsQ, RpsK, and RpsM, were less abundant. This pattern is consistent with the reduced translational capacity inferred from GO enrichment analysis. Interestingly, several proteins in the ESX secretion machinery, including EccA3, EccC3, and EccD3, were also less abundant in the *Δrv0148* mutant, suggesting a role for Rv0148 in the bacterial secretion system as well as host–pathogen interaction pathways.
(d) Stress Response Network: Nineteen proteins within the stress response network, including the metabolic enzymes GltA2 and DapA, were more abundant by more than twofold in the *Δrv0148* strain. Conversely, 26 proteins involved in stress adaptation, including Tpx, GroEL2, SuhB, and Lsr2, were more than two times less abundant (Figure 6D). This mixed pattern suggests potential loss of specific protective mechanisms against oxidative and environmental stresses, alongside simultaneous activation of compensatory pathways that may partially mitigate these deficiencies in the *Δrv0148* strain. However, these suggestions should be validated by further functional assays.
(e) Peptidoglycan Metabolism Network: Peptidoglycan plays a critical role in maintaining *Mtb* cell wall integrity and viability. Given the close relationship between cell wall dynamics, secretion systems, and stress adaptation, we examined SSDAPs associated with peptidoglycan metabolism. Among the proteins in this network, 26 were more and 25 were less abundant by more than two times in *Δrv0148* (Figure 6E). Notably, RpoA, Acn, Eno, and RpsC, which have broader and indirect roles in peptidoglycan metabolism, were more abundant, whereas AcpM was more than ten times less abundant in the mutant.

These findings indicate extensive perturbation of pathways associated with cell wall biosynthesis, stress adaptation, and Type VII secretion in the mutant strain. Collectively, the data suggests that SSDAPs in *rv0148* potentially affects the coordinated regulation of cell envelope maintenance and adaptive responses, ultimately impacting the bacterial fitness and virulence. However, additional functional assays are warranted to confirm and validate these suggestions.

## 3. Discussion

This exploratory proteomic study provides an initial assessment of the effects of Rv0148 deletion on the overall protein content of *Mtb*. Using high-resolution label-free quantitative proteomics, we identified 939 proteins across the WT and *Δrv0148* strains, of which a subset exhibited differential abundance between the two strains. These changes were observed across multiple functional categories, including stress adaptation, intermediary metabolism, secretion systems, redox-associated processes, and cell-envelope-associated functions. Collectively, these findings suggest that deletion of *rv0148* is associated with broad proteomic alterations that may influence bacterial adaptation to environmental stress. Given that Rv0148 belongs to the short-chain dehydrogenase/reductase (SDR) family, whose members are implicated in cellular redox-related processes and intracellular survival in mycobacteria, the observed proteomic changes may reflect interconnected physiological responses to loss of this protein in the mutant [15,16,17]. However, the present study did not directly measure redox status or virulence, and therefore mechanistic relationships remain to be established.

Several proteins involved in protein quality control and stress adaptation exhibited altered abundance in *Δrv0148*. These included GroEL1, GroES, DnaK, and ClpB. Such proteins are well-established components of the mycobacterial proteostasis network and are frequently associated with responses to environmental stress conditions, including oxidative, thermal, and chemical stress [23,24,25,26,27,28]. The coordinated changes observed in these chaperones may indicate an adaptive response to physiological perturbations associated with deletion of rv0148. However, the present proteomic data does not directly demonstrate protein misfolding, aggregation, or proteotoxic stress. Therefore, while the altered abundance of these chaperones is consistent with activation of stress response pathways, conclusions regarding proteotoxic stress or increased proteostatic burden should be considered hypothetical and require validation through complementary functional studies such as aggregation assays, stress challenge experiments, or immunoblot analysis of chaperone expression.

Altered abundance was also observed for several proteins associated with the ESX-3 secretion system, including EccA3, EccB3, EccC3, and EccD3. ESX-3 is known to contribute to metal acquisition and adaptation to nutrient-limited environments and plays important roles in mycobacterial physiology [29,30,31,32]. The differential abundance of multiple ESX-3-associated proteins in *Δrv0148* suggests that deletion of Rv0148 may influence secretion-associated pathways or cellular processes linked to metal homeostasis. However, protein abundance data alone cannot establish direct regulatory interactions or define a regulatory hierarchy. Consequently, the possibility that Rv0148 influences ESX-associated pathways through redox-responsive regulators such as WhiB proteins remains speculative and requires further investigation. Similarly, any potential consequences for host–pathogen interactions, intracellular survival, or virulence should be regarded as hypotheses that need validation through secretion assays and infection-based studies.

The proteomic analysis also identified changes in proteins associated with intermediary metabolism, iron homeostasis, and cell envelope biogenesis. Increased abundance of IdeR and MetE may reflect adaptive metabolic responses in *Δrv0148*, potentially involving pathways related to iron utilization and one-carbon metabolism [31,32]. However, protein abundance alone does not necessarily indicate altered IdeR activity or changes in intracellular iron homeostasis. Direct measurements of intracellular iron levels, expression of IdeR-regulated targets, and transcriptional responses would be required to evaluate this possibility. Similarly, reduced abundance of FadD32 and FbpC suggests potential effects on pathways involved in cell envelope biosynthesis [33,34]. However, because cell wall integrity and virulence were not directly examined in this proteomic experiment, these observations should not be interpreted as direct evidence of impaired cell wall structure or reduced virulence. The reduced abundance of Lsr2, Tkt, and several ribosomal proteins further suggests broader alterations in metabolic and translational pathways that may contribute to the physiological adaptation of the mutant strain [35,36].

The proteomic analysis revealed altered abundance of several proteins involved in oxidative stress defense, including AhpC and KatG. Although both proteins contribute to protection against oxidative stress, they perform distinct biochemical functions. AhpC is a member of the peroxiredoxin/alkyl hydroperoxide reductase family and functions primarily as a thiol-dependent peroxidase involved in the detoxification of organic hydroperoxides, hydrogen peroxide, and reactive nitrogen species-derived oxidants [37,38]. Previous studies have shown that disruption of *ahpC* increases susceptibility to organic hydroperoxides, supporting a role in protection against lipid-derived and alkyl peroxide stress [38,39,40]. Therefore, reduced AhpC abundance could potentially diminish resistance to these oxidative challenges. In contrast, KatG is a bifunctional catalase–peroxidase that contributes to hydrogen peroxide detoxification and is also required for activation of isoniazid [41,42]. Consequently, increased KatG abundance may have implications for both oxidative stress responses and susceptibility to isoniazid. However, changes in protein abundance do not necessarily correspond to altered enzyme activity. Therefore, any compensatory role of increased KatG expression in mitigating oxidative damage should be considered a hypothesis until supported by direct functional measurements.

Taken together, the reciprocal abundance patterns of AhpC and KatG may indicate altered responses of *Δrv0148* to different oxidative challenges. Increased KatG abundance may be associated with enhanced detoxification of hydrogen peroxide, whereas reduced AhpC abundance may decrease protection against organic peroxides and related oxidants [37,38,39,40,41,42,43]. Importantly, these interpretations are based on protein abundance measurements rather than direct functional assays. Nevertheless, the observed proteomic changes are consistent with our previous studies showing increased susceptibility of *Δrv0148* to ROS- and RNS-generating conditions in vitro [16]. Therefore, the current findings provide proteomic observations that are compatible with, but do not directly establish, the mechanisms underlying the previously reported phenotype.

The altered abundance of oxidative stress-associated proteins raises the possibility that deletion of Rv0148 may influence redox-related pathways in *M. tuberculosis*. However, the current study did not measure intracellular redox status, NADH/NAD+ ratios, thiol redox-buffering capacity, oxidation states of iron–sulfur clusters, or the activity of redox-responsive regulators [44,45,46,47,48,49,50]. Consequently, any mechanistic model linking Rv0148 to redox homeostasis remains hypothetical. Similarly, although regulators such as MosR and members of the WhiB family play important roles in redox-responsive gene regulation [44,47,48,49,50,51], the present dataset does not provide direct evidence that Rv0148 interacts with these regulatory pathways. Reduced abundance of PknG may indicate broader physiological changes in the mutant strain; however, protein abundance data alone do not establish altered signaling activity, intracellular survival mechanisms, or redox regulation [52,53]. Future biochemical and genetic studies will be required to define whether Rv0148 participates directly or indirectly in redox-responsive regulatory networks.

The *Δrv0148* mutant also exhibited altered abundance of several proteins involved in stress adaptation, cell envelope-associated processes, and intermediary metabolism. For example, reduced abundance of SigH, MmaA2, HbhA, and LpqE may be associated with changes in stress responses, lipid modification, surface interactions, or envelope-associated functions [54,55,56,57]. However, the present proteomic data do not directly demonstrate impaired capsule remodeling, immune evasion, persistence, or antibiotic tolerance. Likewise, reduced abundance of GarA, MetK, and GlcB suggests potential alterations in metabolic pathways that contribute to bacterial adaptation and survival during nutrient limitation [58,59,60], although direct effects on cell-wall biosynthesis were not evaluated in this study. Conversely, increased abundance of KasB, MetE, AhcY, and GcvP may reflect compensatory metabolic responses aimed at maintaining cellular homeostasis [61,62,63]. Overall, these findings suggest broad physiological adaptation to loss of Rv0148 rather than direct evidence of specific envelope or persistence phenotypes.

Proteins associated with cell envelope maintenance and remodeling also exhibited altered abundance in *Δrv0148*. Increased abundance of FhaA and IniA may be consistent with responses to cell envelope stress or remodeling processes [64,65,66,67,68]. However, the current study did not assess peptidoglycan composition, cross-linking, cell wall permeability, lysozyme susceptibility, cell morphology, or antibiotic susceptibility in the same experimental setting. Therefore, these observations should not be interpreted as direct evidence of enhanced cell wall repair or altered structural integrity. Previous studies from our group demonstrated altered susceptibility of *Δrv0148* to host-associated stresses and antimicrobial challenges [15,16], and the present proteomic findings may provide additional context for those observations. We also observed reduced abundance of SigF, which is important for *M. tuberculosis* stress adaptation and persistence. The biological significance of this change remains unclear and warrants further investigation. Collectively, these findings suggest potential alterations in envelope-associated adaptive responses but require functional validation.

Overall, this exploratory proteomic analysis demonstrates that deletion of Rv0148 is associated with differential abundance of proteins involved in stress adaptation, redox-associated processes, secretion systems, intermediary metabolism, translation, and cell envelope-related functions. Based on these observations, we propose a hypothesis-generating framework in which Rv0148 may influence interconnected redox, stress response, secretion, translational, and envelope-associated pathways. However, the sequence of events linking these proteomic changes to previously reported phenotypes has not been directly demonstrated. Consequently, the proposed relationships between altered redox-associated proteins, stress responses, secretion-associated pathways, cell envelope functions, and attenuation should be considered working hypotheses requiring further validation. Future studies incorporating complementation analysis, targeted biochemical assays, transcriptional profiling, secretion assays, and infection models will be necessary to establish the mechanistic role of Rv0148 in *M. tuberculosis* physiology and pathogenesis.

A key limitation of this study is that the proteomic dataset was generated from pooled triplicate samples, which do not represent the heterogeneity among the different samples. Consequently, the findings herein should be considered preliminary and exploratory. In addition, proteomic analyses quantify protein abundance but do not directly measure protein activity, regulatory interactions, post-translational modifications, or pathway function. Therefore, the observed associations should not be interpreted as evidence of causal mechanisms. The dataset may also underrepresent low-abundance, transiently expressed, or highly regulated proteins. Another limitation is that a complemented *Δrv0148* strain was not included in the proteomic analysis. As a result, it is not possible to attribute all observed changes exclusively to loss of Rv0148 with the same level of confidence that would be provided by genetic complementation. Although complementation of *Δrv0148* was evaluated in our previous phenotypic studies [15,16], inclusion of a complemented strain in future proteomic analyses would strengthen the interpretation of the observed protein abundance changes. Finally, several hypotheses proposed in this study, including potential roles of Rv0148 in redox regulation, secretion-associated pathways, and stress adaptation, remain speculative because the corresponding biochemical and functional mechanisms were not directly examined. Future studies incorporating larger sample sizes, biological replicates, complementation analyses, targeted biochemical assays, and functional validation experiments will be necessary to establish the biological significance of the proteomic changes observed in *Δrv0148*.

## 4. Materials and Methods

### 4.1. Bacterial Strains, Culture Conditions, and Chemicals

Three colonies each of *Mtb* H37Rv (WT) and *Δrv0148* strains were grown in three independent separate flasks to mid-log phase (OD600~0.8) at 37 °C in Middlebrook 7H9 broth (BD Difco^TM^, Franklin Lakes, NJ, USA) supplemented with 0.2% glycerol, 0.05% polysorbate 80, and OADC (oleic acid, bovine albumin, dextrose, and catalase) (BD Difco^TM^, Franklin Lakes, NJ, USA). The *Δrv0148* strain was created and validated in our previous studies as described [15]. Hygromycin (50 μg/mL) as a selective marker was added in the broth to grow the *Δrv0148* strain. All chemicals and reagents used in this study were purchased from Millipore-Sigma (Sigma-Aldrich, Inc., St. Louis, MO, USA) until specified otherwise.

### 4.2. Protein Extraction and Precipitation

Mid-log phase cultures of WT and *Δrv0148* strains were centrifuged at 4000× *g* for 10 min at 4 °C, and the bacterial pellets were washed once with ice-cold phosphate-buffered saline (PBS) to remove residual media components and resuspended in ice-cold lysis buffer containing 50 mM Tris (pH 8), 20% glycerol, 300 mM NaCl, 10 mM imidazole, 0.5 mM phenyl methyl sulphonyl fluoride (PMSF) and a protease inhibitor cocktail. The bacterial suspension was kept on ice and sonicated using an ultrasonic homogenizer at 70% amplitude for 28–30 cycles, with each cycle consisting of a 1 min pulse followed by a 1 min interval. The resultant lysate was centrifuged at 10,000× *g* for 30 min at 4 °C to remove the cell debris and unbroken cells. The supernatant containing whole-cell lysate was transferred to a fresh tube and subjected to protein precipitation using 5% trichloroacetic acid (TCA) as follows. In a 1.5 mL microcentrifuge tube, 1 mL of the whole-cell lysate was mixed with 250 µL of 100% TCA and vortexed vigorously for 1 min to ensure thorough mixing. Samples were incubated overnight at 4 °C to allow protein precipitation. The samples were centrifuged at 14,000× *g* for 10 min at 4 °C, and the supernatant was carefully discarded. The pellet was washed twice with 250 µL of cold acetone to remove residual TCA and other contaminants. Finally, the protein pellet was air-dried at room temperature for 5 min and reconstituted in sterile distilled water to remove residual TCA contaminants. The protein concentration was determined using a bicinchoninic acid (BCA) assay kit, following the manufacturer’s protocol (Thermo Fisher Scientific, Waltham, MA, USA). The integrity and yield of extracted protein were confirmed by SDS-PAGE prior to downstream processing.

### 4.3. SDS-PAGE and Gel Processing

To determine the integrity and quality, 20 μg of extracted protein per sample was resolved on 12.5% SDS-PAGE gels. The gels were incubated for 3 h with a staining solution containing 0.25% Coomassie blue R-250, 40% methanol, 10% glacial acetic acid, and 50% distilled water. The stained gels were destained using a solution composed of 45% methanol, 10% glacial acetic acid, and 45% water. The destaining solution was changed periodically until the Coomassie blue was completely removed from the gels. Finally, the gels were rinsed with water, and protein bands of interest were excised carefully using a sterile scalpel. The excised gel pieces were diced into ~1 mm^3^ cubes, transferred into 1.5 mL microcentrifuge tubes, and washed with Milli-Q water to remove residual stains and contaminants. Briefly, the gel fragments were vortexed at 1500× *g* for 1 h, followed by removal of the wash solution and a repeat wash under identical conditions. After washing, the gel pieces were spun briefly at 3000× *g* for 20 s, and the supernatant was carefully discarded. Subsequently, 300 µL of a 1:1 (*v*/*v*) mixture of 100 mM ammonium bicarbonate and acetonitrile was added to each tube, followed by vortexing at 1500× *g* for 30 min to further de-stain the gel pieces. This was followed by the addition of 500 µL of 100% acetonitrile and incubation at room temperature with gentle vortexing until the gel pieces turned transparent. The acetonitrile was completely removed, and tubes were left open for 20 min to allow residual solvent to evaporate. The gel pieces were stored at −20 °C.

### 4.4. In-Gel Trypsin Digestion and Peptide Extraction

Excised gel pieces of ~500 ng to 2 μg were destained using ammonium bicarbonate and acetonitrile. Carbamidomethylation of cysteines (+57.0215 Da) was verified as a fixed modification resulting from standard experimental alkylation; proteins within the destained gel matrix were reduced with 10 mM dithiothreitol at 56 °C and alkylated with 20 mM iodoacetamide incubation at room temperature for 30 min. For enzymatic digestion, the gel pieces were rehydrated with 50 µL of sequencing-grade trypsin solution (13 ng/µL in 50 mM ammonium bicarbonate) and incubated on ice for 30 min. The trypsin-to-protein ratio was 1:30. The buffer volume was checked and, if needed, additional trypsin buffer was added to ensure complete rehydration of the gel pieces. After absorption of the enzyme solution into the gel matrix, the samples were incubated overnight at 37 °C with gentle shaking at 300× *g* to facilitate protein digestion. Following digestion, the trypsin-containing supernatant was carefully transferred to microcentrifuge tubes. To extract remaining peptides from the gel, an extraction buffer (volume approximately twice that of the trypsin buffer, typically 100 µL) was added to the gel pieces (followed by vigorous vortexing for 20 min) along with 50% acetonitrile. The combined extracts were dried completely using a SpeedVac concentrator, desalted, reconstituted in 30 µL of 3% acetonitrile, and stored at −20 °C.

### 4.5. Liquid Chromatography–Mass Spectrometry (LC-MS)

The peptides were analyzed in duplicate using a nano ACQUITY UPLC chromatographic system (Waters, Manchester, UK) equipped with MassLynx4.1 SCN781 software for data acquisition. Chromatographic separation was performed using a Symmetry^®^ C18 trap column (180 μm × 20 mm, 5 μm) and an HSS T3 C18 analytical column (75 μm × 200 mm, 1.8 μm). (Waters, Manchester, UK). The experiment was performed with two technical replicates. The separation was carried out in reverse-phase mode, with a mobile phase consisting of solvent A (0.1% formic acid in water) and solvent B (0.1% formic acid in acetonitrile). The gradient program was as follows. It began with 99% solvent A and 1% solvent B, held for 3 min. The composition was then linearly changed to 60% solvent A and 40% solvent B by 43 min, and further to 20% solvent A and 80% solvent B by 46 min, which was maintained until 50 min. The gradient was then returned to the initial conditions of 99% solvent A and 1% solvent B for 51 min and held for 60 min. The total run time was 60 min, and the flow rate was set at 300 nL/min, with the column temperature maintained at 35 °C and the autosampler temperature at 4 °C, with a final concentration of 0.1% formic acid. The peptides eluted from the chromatography column were analyzed through mass spectrometry (MS).

### 4.6. Mass Spectrometry Acquisition and Protein Identification

All the MS runs were performed using ion mobility-enabled separation on a Synapt G2 High-Definition MS^TM^ System (HDMSE System; Waters, Manchester, UK). Data acquisition was conducted positively, with a nano-electrospray ionization (nano-ESI) capillary voltage set at 3.4 kV. The sample cone voltage was 40 V, and the extraction cone voltage was 4 V. The ion mobility spectrometry (IMS) gas (N2) flow was maintained at 90 mL/min. The IMS T-Wave^TM^ pulse height was set at 40 V, and the IMS T-Wave^TM^ velocity was 800 m/s, with IMS ramping between 8 V and 20 V. The system operated in resolution mode, and data was acquired in a continuum format. The collision energy was ramped from 20 eV to 45 eV. To ensure system stability, column equilibration was performed before each injection, and regular blanks were run to maintain carryover at <0.1%. Raw MS data was processed using Progenesis QI for Proteomics V4.2 (Non-Linear Dynamics, Waters) as described previously [69,70]. Protein identification was performed using *Mtb* databases sourced from UniProt (https://www.uniprot.org/) reviewed entries (retrieved on 17 July 2026). The identification criteria were set at ≥1 peptide per protein, with peptide assignments supported by fragment ion evidence (≥1 fragment per peptide and ≥3 fragment ions per protein). Cysteine carbamidomethylation was considered a fixed modification, while methionine oxidation was treated as a variable modification. For each identified and annotated *Mtb* protein, we determined the normalized expression abundance value using a cutoff false discovery rate (FDR) of 4%, allowing for 1 missed cleavage.

### 4.7. Proteomics Data Analysis

Based on the MS data on identified *Mtb* proteins with maximum normalized abundance, protein identity was established from the UniProt-formatted description field of each mass-spectrometric identification and matched by protein name across technical replicates, as accession groupings assigned during database searching varied slightly between replicates of the same protein. Within a different gel sample of same strain, duplicate entries corresponding to the same protein were resolved by retaining the maximum normalized abundance, and proteins not detected in each replicate were assigned a value of zero. For each protein, the maximum normalized abundance across all ten mass spectrometry measurements (five Gel samples × two technical replicates) was taken as the representative abundance value for comparison between WT and *Δrv0148* mutant groups. Gene symbols and Mycobacterium tuberculosis H37Rv reference accessions (UniProt, NCBI taxonomy ID 83332) were assigned from the corresponding UniProt entry and confirmed by direct query of the UniProt database. Proteins with expression ratios >2 were classified as upregulated, and those with ratios <0.5 were considered downregulated in *Δrv0148* compared to the WT strain.

Functional enrichment and classification of SSDAP proteins was performed using ShinyGO (v0.75), against the Gene Ontology (GO) biological processes, cellular compartment, and molecular function databases. Statistical significance was calculated using a hypergeometric test, which evaluated the number of functionally related query proteins against the total background genes, with the default *Mtb* genome (H37Rv) as the reference background, with an FDR cutoff <0.05. Further, STRING 12.0 was employed to cluster the upregulated and downregulated proteins based on predicted functional protein–protein interactions (PPI) and categorize them based on functional roles. Finally, GraphPad Prism 10 was used to visualize the expression patterns, highlighting proteins involved in pathways that were distinctly upregulated and/or downregulated in WT and/or *Δrv0148* strains. The raw and processed proteomics data was submitted to the MassIVE repository of the USA-NIH funded Center for Computational Mass Spectrometry (Accession# MSV000102199). It should be noted that datasets submitted to MassIVE are consistent with ProteomeXchange policies for publication requirements. A list of most and least abundant proteins used for ShinyGo analysis is provided in Supplementary Table S3.

### 4.8. Statistical Analysis

The proteomics analysis was performed with three pooled samples per *Mtb* strain in two technical replicates. Liquid cultures of three independent colonies of WT and *Δrv0148* strains were pooled and used as two technical replicates for proteomics analysis. The average values were taken for further analysis. The LC-MS data was processed with Progenesis v 3.0 QI output software (Waters Corporation, Milford, MA, USA)-based alignment and normalized across samples. GraphPad Prism-10 (GraphPad Software, Boston, MA, USA) was used to plot heat maps.

## Data Availability

The processed data generated and/or analyzed during the current study are presented in Supplementary Tables S1–S3. The raw MS data were deposited in the public repository.

## Supplementary Materials

The following supporting information can be downloaded at: https://www.mdpi.com/article/10.3390/ijms27188318/s1.
