# Supplementary material for: A Pilot Proteomics Analysis of Mycobacterium tuberculosis Lacking a Putative Short-Chain Dehydrogenase Rv0148
Source: Int J Mol Sci. 2026 Sep 18;27(18):8318. doi: 10.3390/ijms27188318 (PMC13607042; doi:10.3390/ijms27188318)
Supplement: Supplementary file 1 [file ijms-27-08318-s001.zip › Supplementary Figure S1.pdf]

Supplementary Figure S1. Network interaction analysis. (**A).** Interaction map of various biological processes comprised of significantly upregulated proteins in *Δrv0148* mutant compared to WT. (**B).** Interaction map of various biological processes comprised of significantly downregulated proteins in *Δrv0148*mutant compared to WT.

A

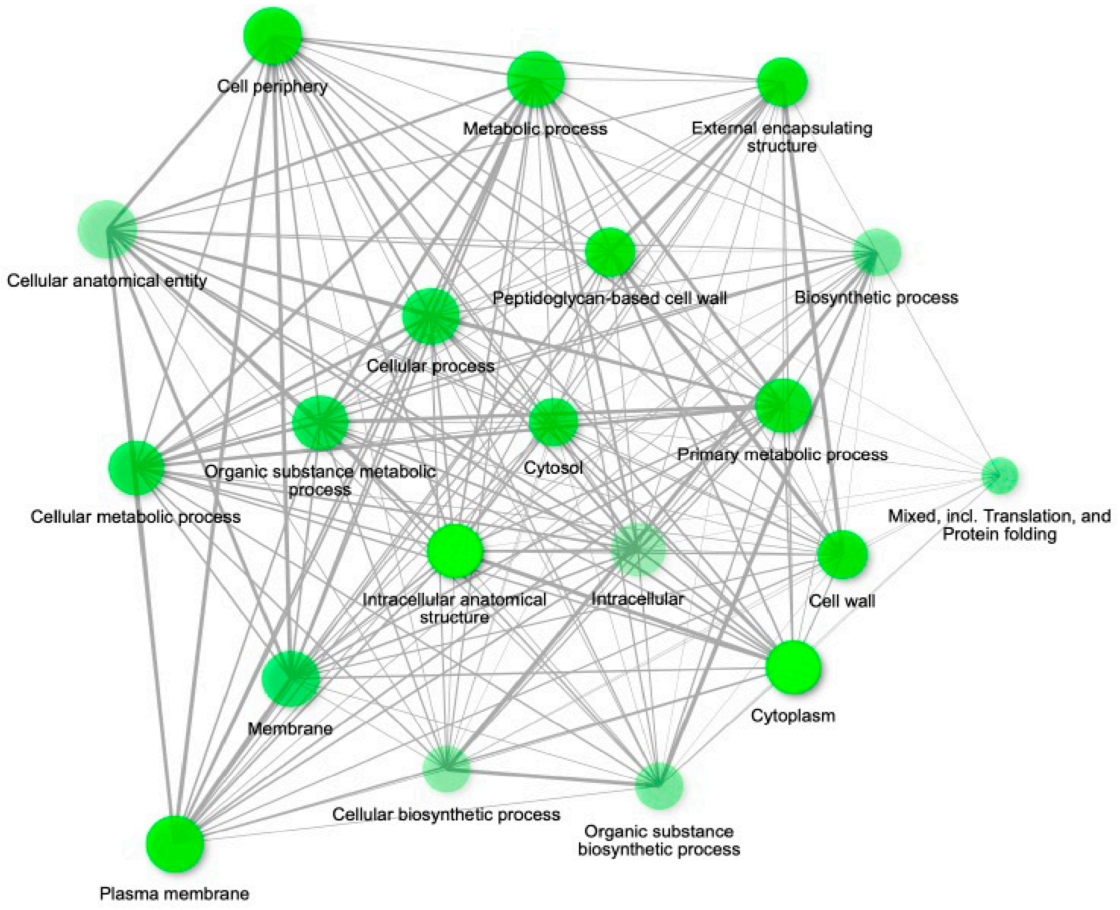

B

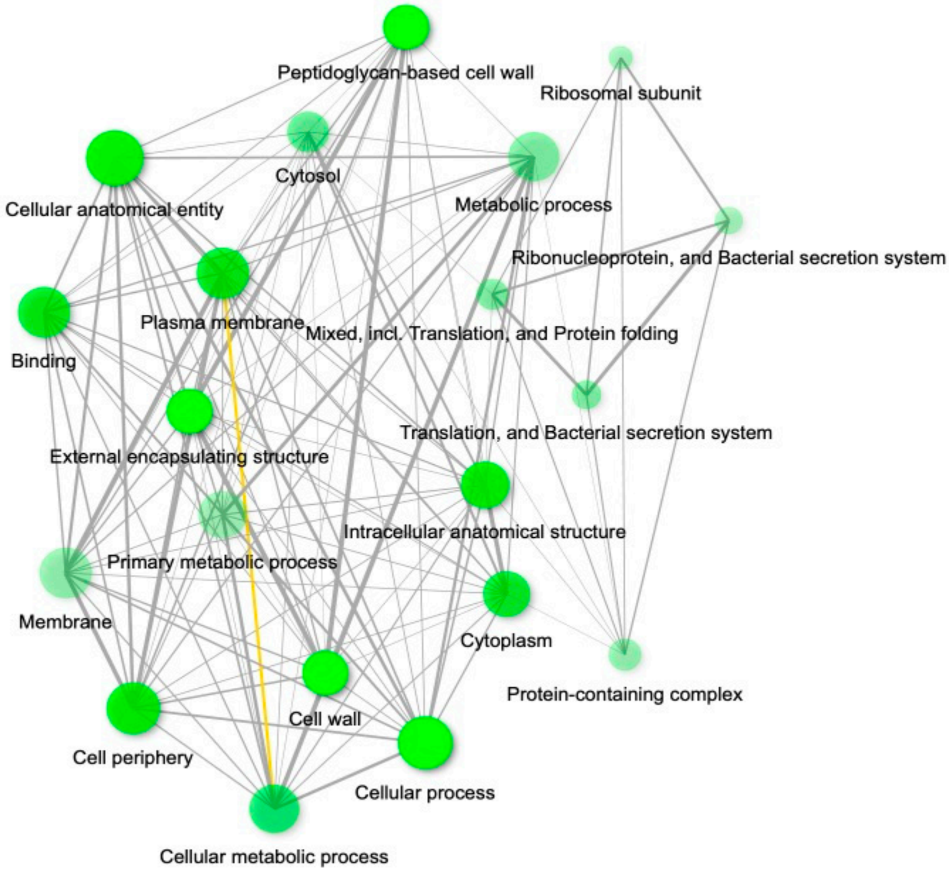

Peptidoglycan-based cell wall
Ribosomal subunit
Cytosol
Cellular anatomical entity
Metabolic process
Ribonucleoprotein, and Bacterial secretion system
Plasma membrane
Mixed, incl. Translation, and Protein folding
Binding
Translation, and Bacterial secretion system
External encapsulating structure
Intracellular anatomical structure
Primary metabolic process
Membrane
Cytoplasm
Protein-containing complex
Cell wall
Cell periphery
Cellular process
Cellular metabolic process
