# Supplementary material for: A Pilot Proteomics Analysis of Mycobacterium tuberculosis Lacking a Putative Short-Chain Dehydrogenase Rv0148
Source: Int J Mol Sci. 2026 Sep 18;27(18):8318. doi: 10.3390/ijms27188318 (PMC13607042; doi:10.3390/ijms27188318)
Supplement: Supplementary file 1 [file ijms-27-08318-s001.zip › Supplementary Figure S2.pdf]

Supplementary Figure S2. STRING analysis of significantly upregulated (**A)** and downregulated (**B**) proteins in *Δrv0148* mutant compared to WT showing the interaction among proteins of different categories shown in different colors.

A

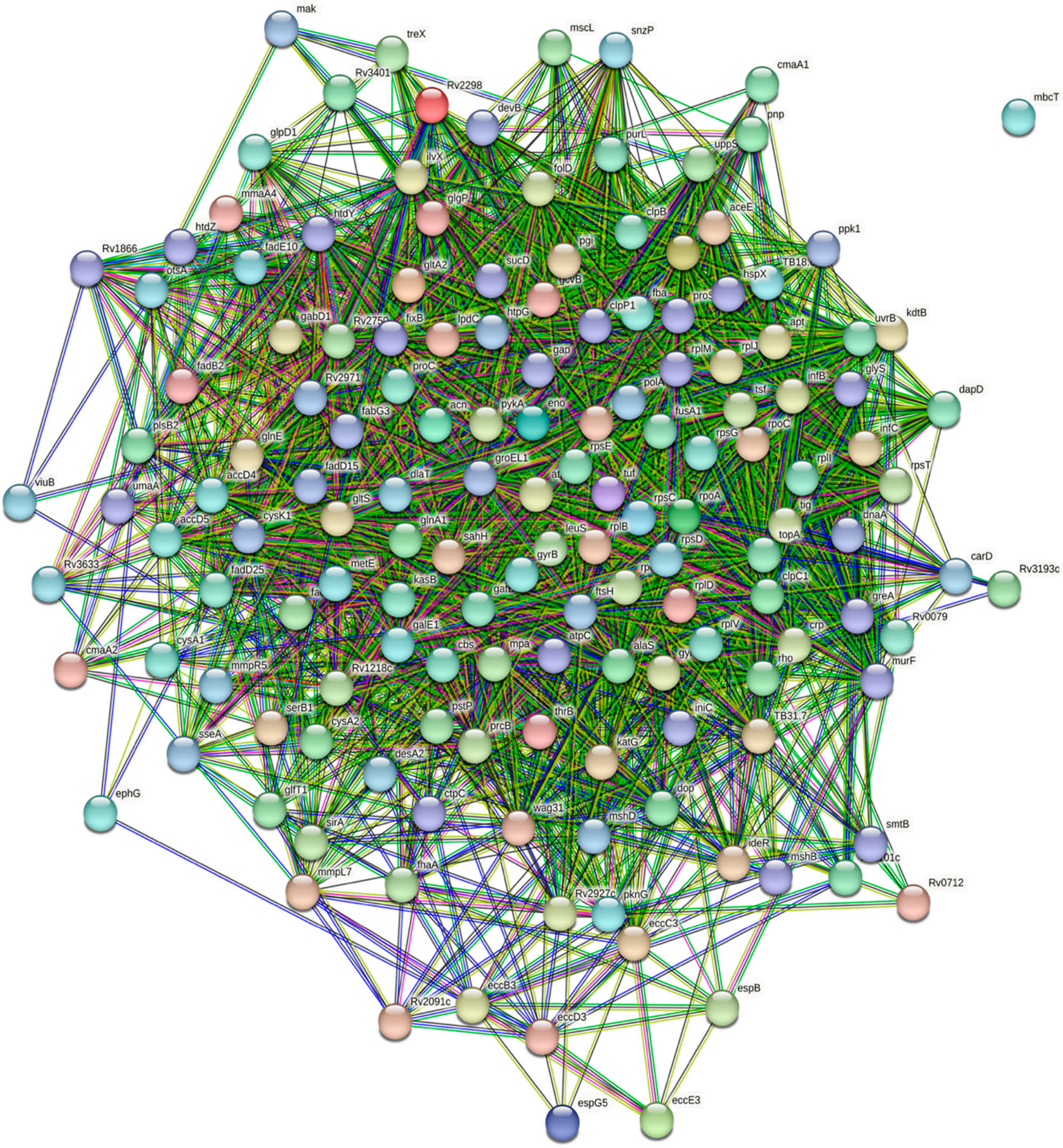

B

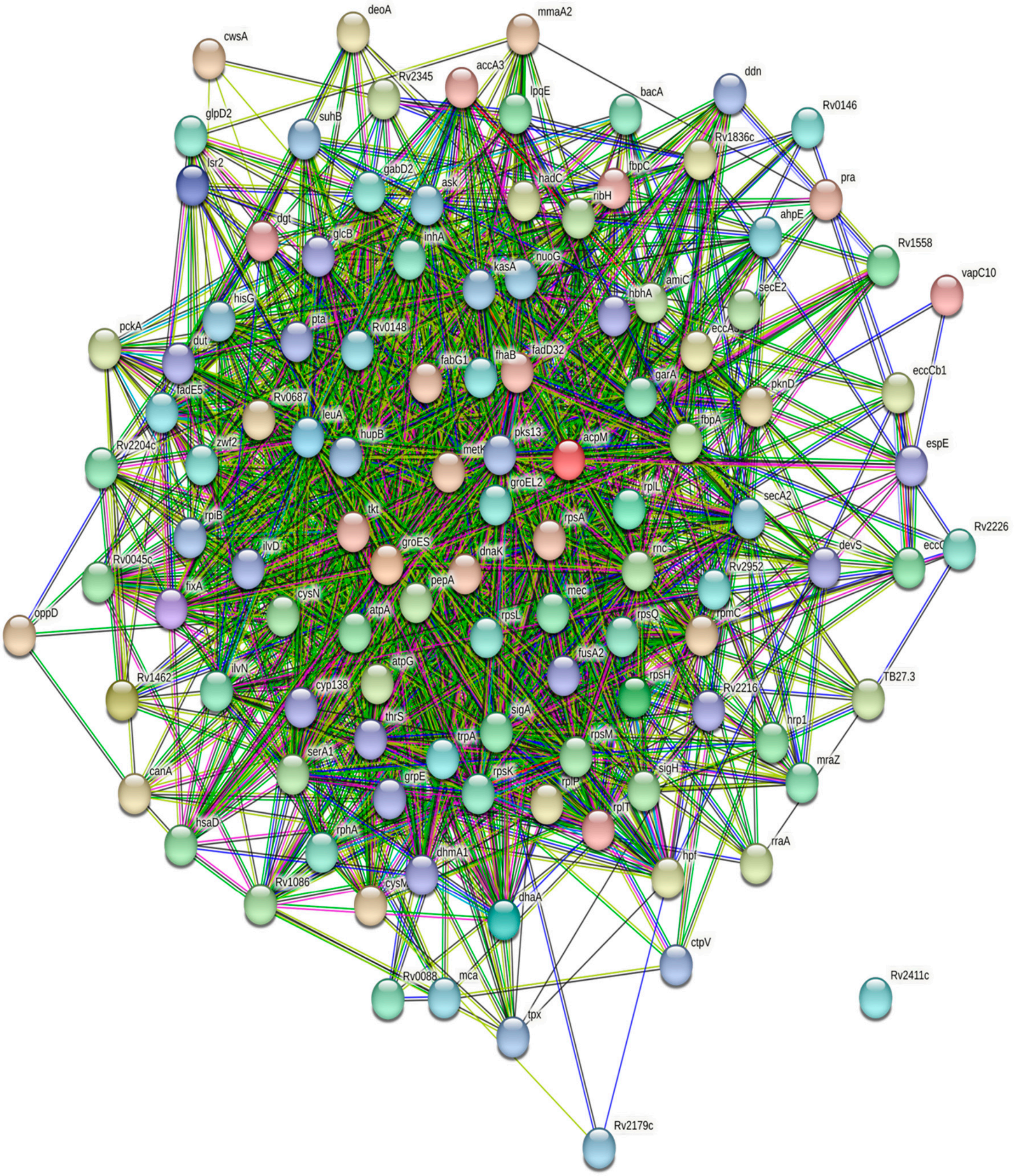

deoA
mmaA2
cwsA
Rv2345
accA3
lpqE
bacA
ddn
Rv0146
glpD2
suhB
Rv1836c
lsr2
gabD2
ask
hadC
fbpC
ribH
pra
ahpE
dgt
glcB
inhA
kasA
nuoG
Rv1558
vapC10
hisG
amiC
hbhA
secE2
pckA
pta
Rv0148
dut
fabG1
fhaB
fadD32
eccCb1
fadE5
garA
pknD
Rv0687
leuA
hupB
fbpA
espE
Rv2204c
zwf2
metK
pks13
acpM
groEL2
rplL
secA2
rpiB
tkt
rpsA
Rv2226
ilvD
groES
dnaK
devS
Rv0045c
rnc
Rv2952
fixA
pepA
cysN
mec
oppD
atpA
rpsL
rpsQ
rpmC
fusA2
Rv1462
ilvN
atpG
rpsH
cyp138
Rv2216
TB27.3
thrS
sigA
hrp1
trpA
rpsM
serA1
canA
grpE
rpsK
rplP
sigH
mraZ
rplT
hsaD
rphA
dhmA1
hpf
rraA
Rv1086
cysM
dhaA
ctpV
Rv0088
mca
Rv2411c
tpx
Rv2179c
